# Gas analysis system for studying sand mold’s atmosphere during steel casting

**DOI:** 10.1016/j.mex.2025.103366

**Published:** 2025-05-10

**Authors:** Jakub Wielgosz, Alexis Vaucheret, Philippe Jacquet, Jean-François Carton

**Affiliations:** a Arts et Metiers Institute of Technology, LaBoMaP, 13 rue Porte de Paris, Cluny 71250, France; b Safe Metal, 1 Boulevard de la Boissonnette 42110 Feurs, France

**Keywords:** Gas analysis, Foundry, Sand casting, Mold-metal interactions, Steel casting, Gas sensors, Foundry Appropriate Gas Analysis System (FAGAS)

## Abstract

The composition of the mold atmosphere plays a critical role in mold-metal interactions during steel casting and is a root cause of certain casting defects. However, studying this atmosphere is challenging due to technical difficulties in extracting gases from sand molds and the harsh conditions of the metal casting process. Most of the studies realized in the field uses gas chromatography and mass spectrometry technics to investigate atmosphere in a foundry mold. Single-gas sensors, being readily available nowadays, present a cost-effective alternative to aforementioned technics. In this paper, we present an innovative gas analysis system designed for real-time, in-situ monitoring of the mold atmosphere. This system has been developed in collaboration between ENSAM Cluny (LaBoMaP) and the industrial partner Safe Metal.

• *The paper presents the design, fabrication, operation and validation of an in-house foundry gas analyzer.*
• *Issues regarding gas sampling from a sand mold are discussed.*
• *The measurements with the analyzer were confronted with a reference gas mixture as well as with the foundry gas samples analyzed by µGC method.*

## Graphical abstract

**Figure:**
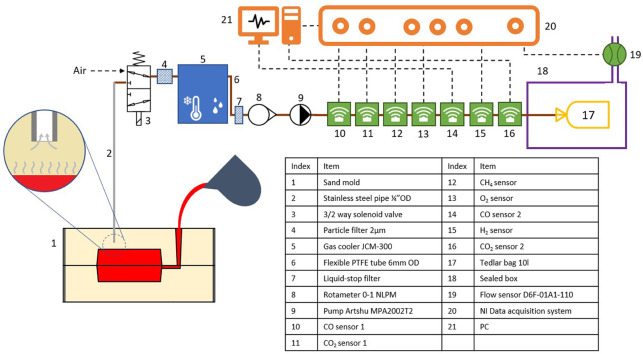

Specifications table

**Table:** 

Subject area:	Engineering
More specific subject area:	Steel Casting
Name of your method:	Foundry Appropriate Gas Analysis System (FAGAS)
Name and reference of original method:	
Resource availability:	*Hardware:* • *Data acquisition:*https://www.ni.com/en/shop.html#pinned-nav-section1 • *Gas filter and cooler:*https://www.smartgas.eu/en/products/accessories • *Sensors details: information on demand* *Software:* • *DASYLab:*https://digilent.com/reference/software/dasylab/start?srsltid=AfmBOoqTOFjk5VOZNPt5vuEbhYrOacU3umKDHnShByKPzFCXsn_Dtxuu

## Background

Sand casting is a very popular manufacturing process for producing ferrous castings, in which the molten metal is poured inside a mold made out of bound sand. When the mold heats up the binder undergoes a decomposition. In case of green sand molds, the water contained in the sand evaporates in a violent manner. In sands bound with organic resin, the products of resin’s combustion and pyrolysis are released within the molding aggregate [1]. Depending on the nature of the released gases they can enter a further chemical reaction with the melt’s or mold’s constituents. The sand mold’s atmosphere during steel casting is primarily composed of H_2_, CO, CO_2_, CH_4_, N_2_ and O_2_ [[2], [3], [4]]. Although other gas species are also present in minor concentration, the gases listed above are the ones of main importance.

The composition of the mold atmosphere is a crucial parameter influencing mold-metal interactions which underlie many of the surface defects of steel castings. Oxidizing atmosphere, which is usually estimated by means of ratio CO_2_/CO, may lead to the casting defect known as chemical metal penetration (or burn-on). The defect starts with oxidation of the casting surface by the mold atmosphere. In contrary to the liquid steel, iron oxide exhibits wetting behavior towards sand. In consequence melt penetrates the interstitials between sand grains resulting in adherent layer of sand [[5], [6], [7]]. High presence of hydrogen or nitrogen, on the other hand, can lead to the solubility gas holes known as pinholes. Diatomic gases tend to dissolve in liquid and solid metal therefore their presence in the mold results in gas pick-up by the melt. During solidification the gas solubility plummets and the gas is ejected from nucleating solid phase into liquid phase. At some point, gas can precipitate from supersaturated liquid and form a bubble in the molten metal leading to the creation of porosity in a casting part [[8], [9], [10]].

The formation of the atmosphere in sand molds is a complex mechanism which is studied scarcely by means of numerical simulation although some attempts have been made [6]. For this reason, the experimental approach is much more common. Several studies focused on the atmosphere in sand molds poured either with cast iron [3,4,7,[11], [12], [13]] or steel [2,4,11,14,15]. Nevertheless, the influence of certain process parameters as well as sand ingredients remains unclear. In most cases, including aforementioned studies, analysis of the gas composition in sand molds was conducted using mass spectrometry (MS) and gas chromatography (GC). Such equipment can be regarded as expensive and its use in the foundry environment is risky due to the high presence of dust and metal spatters.

This paper presents the design, fabrication, operation and validation of the Foundry Appropriate Gas Analysis System (FAGAS) dedicated to studying the sand mold’s atmosphere. The system uses single-gas sensors, that is sensors measuring concentration of one gas at a time, which collectively provide a complete analysis of the gas composition. Those sensors, often of industrial use, are much less expensive than GC or MS equipment and can be regarded as low-maintenance. The potential damage to such a sensor is less costly and replacement much easier.

## Method details

### Design and fabrication

The presented system has been developed at ENSAM Cluny (LaBoMaP) in collaboration with the industrial partner – Safe Metal. It should be noted that as far the laboratory was not equipped with any scientific instrument enabling quantitative analysis of the gas composition. The idea behind the development was to use off-the-shelf gas sensors to create a gas analyzer, appropriate to foundry, to enable the study of the gas composition on site. Following design assumptions about the system were made at the beginning of the project:

• Mobility – the system needs to be moved around easily so that it does not stay in the foundry laboratory permanently,
• Capability to quantify gases which are known to be the main constituents of the mold’s atmosphere (H_2_, CO, CO_2_, CH_4_ and O_2_),
• Capacity to acquire analog signal from external instruments such as pressure transducers and thermocouples,
• Capacity of continuous online gas measurement during casting.
• System must be adapted to the sand-casting process by proper conditioning of the sample prior to the analysis.
• Ability to control and regulate flowrate during gas extraction.

**Fig. 1:**
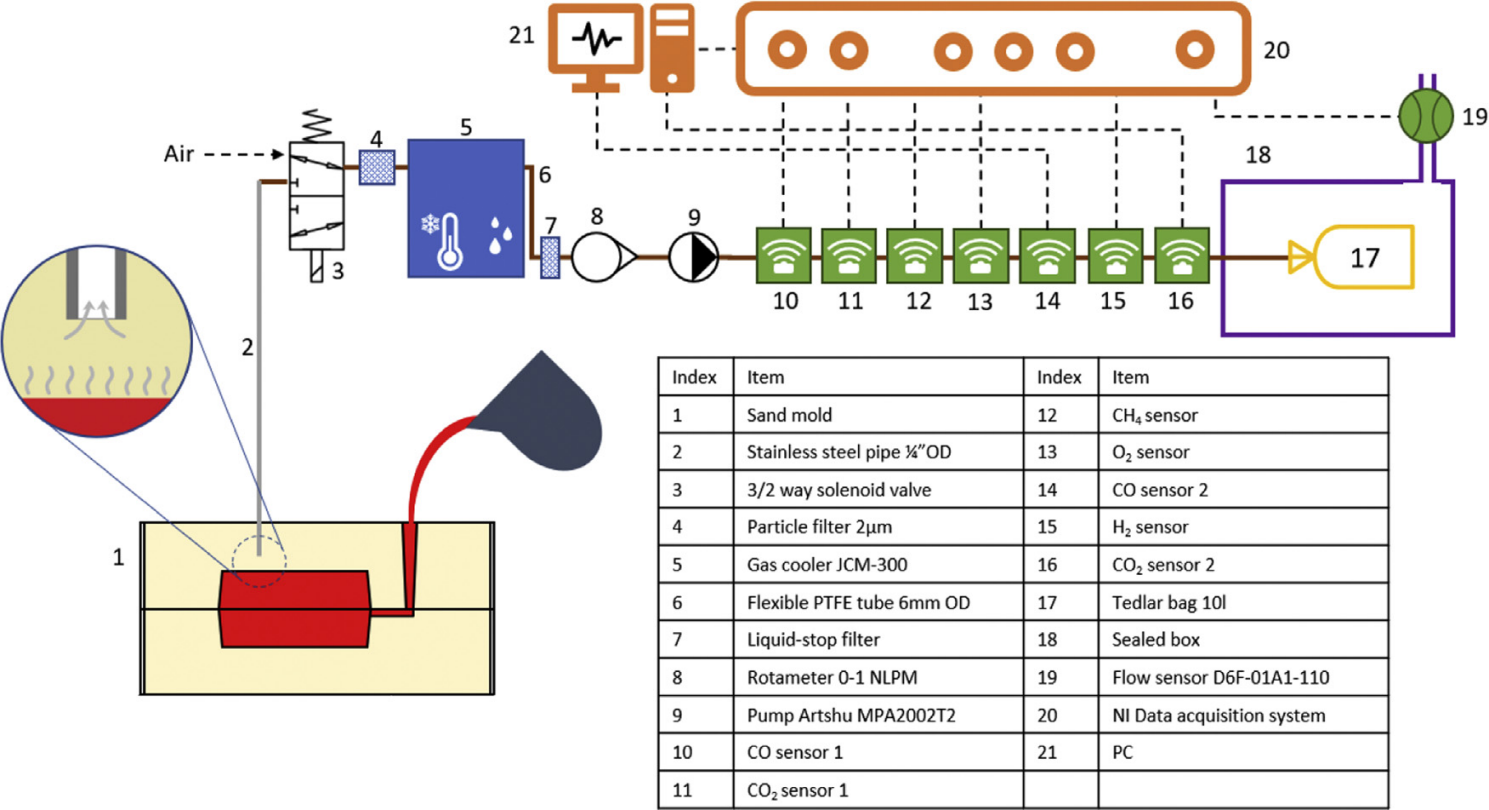
Scheme of the foundry appropriate gas analysis system (FAGAS). Some elements of the pneumatic system, power supply and electronics were not included in the schematics.

The concept of the system is presented in a form of a pictographic scheme in Fig. 1. Elements in blue (4, 5, 7) are responsible for sample conditioning. Sensing elements (20, 21) are in green while data acquisition components are in orange. Components in black color (3, 8, 9) are responsible for the flow control.

The system works in the following way: The gas is drawn from the mold (1) using a diaphragm pump (9) via a stainless-steel tube inserted in the sand (2). The gas sampling starts by switching the position of the 3/2 solenoid valve (3). The gas first passes through a 2 µm particle filter (4) so that fines drawn from the sand mold with gas are removed. In case of green sand, the water vapor is highly present in the mold resulting in the wet gas. Since the sensors cannot work under humidity exceeding 100 %RH the gas is cooled in the gas cooler (5) where condensate is removed via a capillary placed at the bottom of the U-tube. Additional element – tube filled with silica gel – may be added in order to increase the drying performance. Afterwards the gas passes through a liquid-stop filter (7) which is a safety measure which clogs in case of liquid condensation preventing the gas from advancing in case of a failure of the gas cooler (5). The flow can be controlled through a Key Instruments FR2000 flowmeter (8) calibrated on air. Single-gas sensors (10–16) measure the concentration of each gas species in the sample. The gas is pumped into a Tedlar bag (17) which is placed inside an air-tight box (18). The only outlet from the box leads via a flow sensor (19) calibrated on air. As the Tedlar bag (17) is filled with gas the air from the box (18) is pushed away via an outlet enabling an indirect flowrate measurement which is not possible using rotameter (8) when the gas is different than the air. Data from all sensors is gathered using a NI data acquisition cart (20) connected to a PC (21) using an USB cable. Acquisition is managed via program prepared in DASYLab software.

**Fig. 2:**
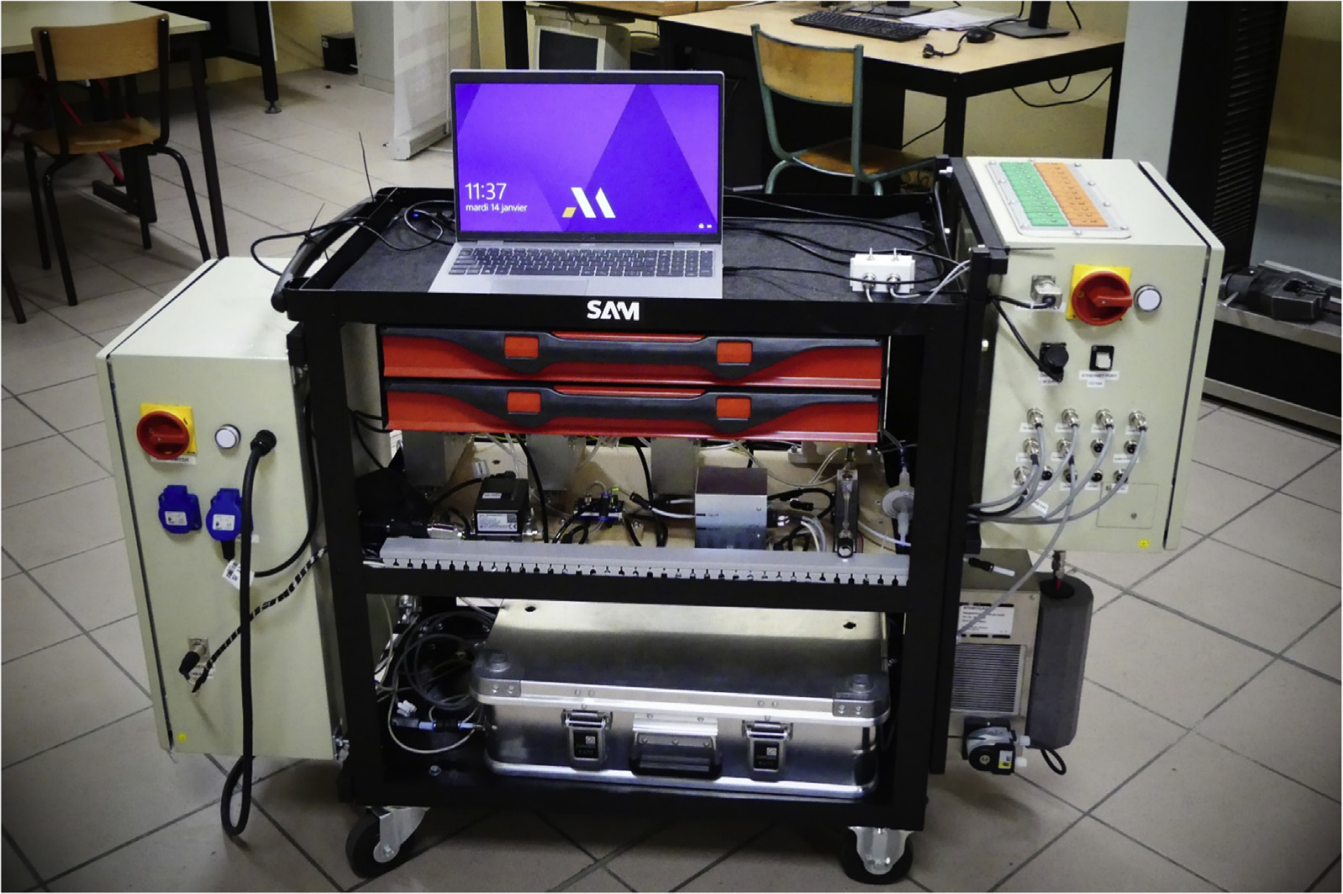
Photography of FAGAS.

To address a mobility requirement, it was decided to build the system on a trolley in order to assure high mobility. SERVI-3P2T tray tool trolley from SAM was chosen as the base. Fig. 2 presents a photo of the FAGAS analyzer. For the sake of clarity same visible components of the system were labeled with the same indexes as in Fig. 1. Upper tier of the trolley serves as a working surface for the operator. The middle tier is serves for sensors, pumps and filters (3, 4, 6–16). Air-tight box is placed in the lower tier (17–19). Additionally, at the sides of the trolley there is a power supply electrical box, data acquisition electrical box (20) and gas cooler (5). Auxiliary tools, documentation and replacement parts are stored in the drawers below upper tier.

### The choice of the sensors

Table 1 present the list of chosen gas sensors. Depending on the gas, different measuring technology can be applied. Non-dispersive infrared sensors were chosen for CO, CO_2_ and CH_4_ since all those gases absorb part of the infrared spectrum. Oxygen is measured with a paramagnetic sensor as it is one of few gases exhibiting paramagnetic properties. Hydrogen is measured with thermal conductivity sensor thanks to its elevated thermal conductivity. Detection ranges were chosen based on availability and concentrations encountered in studies performed at the University of Alabama [14]. To address the issue of online gas measurement the response time of gas sensors should be as low as possible. Nonetheless two sensors with relatively high response time were added as their fast counterparts presented deviations in the measurement. This will be demonstrated in the method validation part.

**Table 1:** Choice of the single-gas sensors.

Target gas	Nomenclature	Technology	Detection range	Response time
H_2_	H_2_ sensor	Thermal conductivity	0 – 100 vol%	t_90_ < 5s
CO_2_	CO_2_ sensor 1	Non-dispersive Infrared	0 – 50 vol%	t_90_ < 3s
CH_4_	CH_4_ sensor	Non-dispersive Infrared	0 – 15 vol%	t_90_ < 3s
CO	CO sensor 1	Non-dispersive Infrared	0 – 100 vol%	t_90_ < 3s
O_2_	O_2_ sensor	Paramagnetic	0 – 25 vol%	t_90_ < 3s
CO	CO sensor 2	Non-dispersive Infrared	0 – 100 vol%	t_90_ < 14s
CO_2_	CO_2_ sensor 2	Non-dispersive Infrared	0 – 100 vol%	t_90_ < 30s

Few things should be noted while choosing a gas sensor. First, sensors might have cross-sensitivity towards gases different than the target gas – caution should be taken while selecting gas sensors. Out of all presented sensors infrared sensors are most prone to cross-sensitivity as the gas absorption spectra often partially overlap. Hydrogen thermal conductivity sensor experiences cross-sensitivity towards helium but this gas is not present in the foundry mold. Also depending on the type of sensor and its design different response times t_90_ are to be expected. Response time of a sensor is increased when the flow is not an ideal-plug flow. Hence presence of elements such as a filter or an orifice render response time longer.

First five sensors in the Table 1 feature a heated measuring cell so that the temperature is kept constant throughout the measurement. O_2_ sensor was equipped with an internal by-pass to ensure a constant flowrate of 150 ml/s through the measuring cell. Additionally, CO_2_ sensor 1, CO sensor 1, CH_4_ sensor, and H_2_ sensor feature pressure compensation and temperature, with the latter performing compensation with respect to the gas humidity as well. During the measurement the gas doesn’t flow directly through measuring cells of H_2_ sensor and CO_2_ sensor 2 so that they were equipped with in-line adapters which may affect their response time.

All sensors, except for O_2_ sensor, are factory-calibrated. O_2_ sensor can be calibrated on air and an oxygen-free gas for span and zero calibration respectively. The calibration is done by adjusting corresponding potentiometers so that the reading matches the actual concentration of the oxygen being supplied.

### Data acquisition

H_2_ sensor, CH_4_ sensor, CO sensors (1 & 2) as well as CO_2_ sensors (1 & 2) feature digital outputs and use Modbus RTU protocol for communication. Furthermore, sensors are equipped with analogue outputs 0–10 V (H_2_ sensor) or 4–20 mA (CO_2_ sensor 1, CO sensor 1, CH_4_ sensor, O_2_ sensor) related linearly to the measured gas concentration. Flowrate sensor D6F-01A1–110 has an analogue output 0–5 V which is related to the reading by means of a quadratic function. NI-9205 card, used with Compact DAQ, featuring 32 analogue input channels (voltage) is used to register analogue signals. Voltage outputs are connected directly to the DAC card whereas the output current is measured indirectly by passing the current through a high precision 500 Ohm resistor (±0.01 %) and measuring the voltage across its terminals. As far as CO sensor 2 and CO_2_ sensor 2 are concerned the sensor readings are acquired from the numerical signal using a USB adapter and dedicated software provided by the manufacturers.

Additionally, the acquisition box hosts 16 thermocouple sockets (8 type K and 8 Type S) for temperature measurement. The acquisition of the signal from thermocouples is realised with two NI-9212 cards each having 8 thermocouple-dedicated channels.

### Conditioning of the gas sample

Sampling gas from a sand mold during steel casting comes with certain issues. Gas drawn from the vicinity of mold-metal interface comes at an elevated temperature. Additionally, large quantities of water vapor can be found in the sample due to its progressive evaporation from the mold. Moreover, fine particles of sand are drawn along with the gas. Finally, harmful gas species such as H_2_S are found due to the sulphur presence in the sand which, although present in small concentration, may react with water vapor to create sulphuric acid. All of the listed issues can potentially damage sensing equipment or lead to false readings. However, the risk can be minimized with a proper conditioning the sampled gas.

First element responsible for sample conditioning is in fact the stainless-steel (SS) tube inserted in the sand. Some installations for sampling foundry gas feature a heated transfer line [13,16]. For example, the test stand, described in Patent PL 224,705 B1, used for measurement of emissions of harmful gases from moulding sands uses a heated transfer line to prevent the condensation of gaseous products before they reach the sorption tubes [17]. Here a difference approach was taken. Condensation temperature of all gases of interest is far below 0 °C therefore there is no point in heating the transfer line. Instead drawn gas is pre-cooled in the transfer line and great part of fines are removed from the gas during condensation of the water. Although it renders tubes full of slurry and require regular changing at the end the solution is cost-effective due to low price of the stainless-steel tubes compared to other components of the system. Residual fines are removed with 2 µm particle filter. Gas is further cooled with JCM-300 gas cooler which uses Peltier effect to remove heat. Condensate is removed with a pump through a capillary placed at the bottom of the cooler. Liquid-stop filter ensures that no condensate advances towards the sensors. The gas is than reheated in measuring cells so that any potential condensation of residual humidity is prevented. The fact that the gas is cooled, dried and then reheated also minimize the risk of producing sulphuric acid.

## Method validation

### Sensor response to the reference gas

**Fig. 3:**
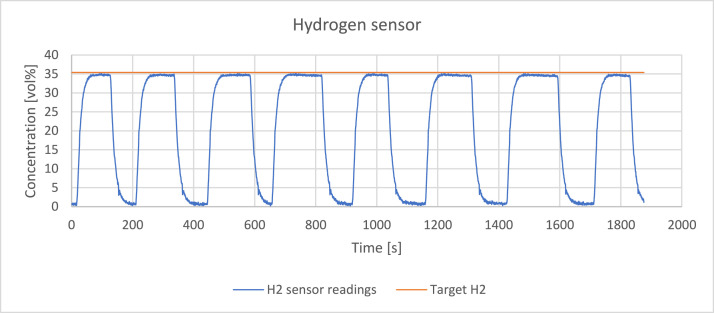
Response of the hydrogen sensor to the reference gas mixture.

**Fig. 4:**
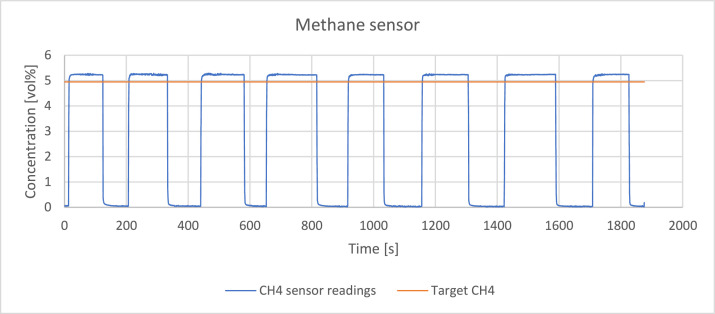
Response of the methane sensor to the reference gas mixture.

**Fig. 5:**
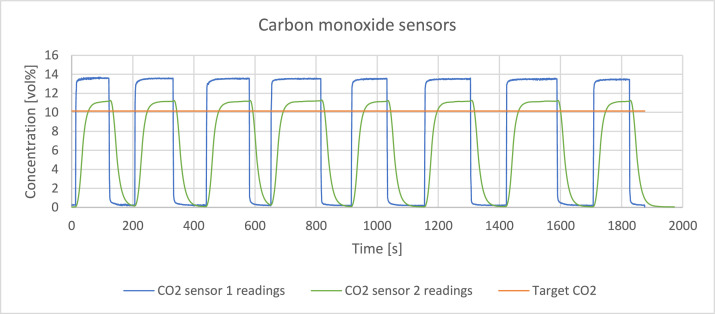
Response of the carbon dioxide sensors to the reference gas mixture.

**Fig. 6:**
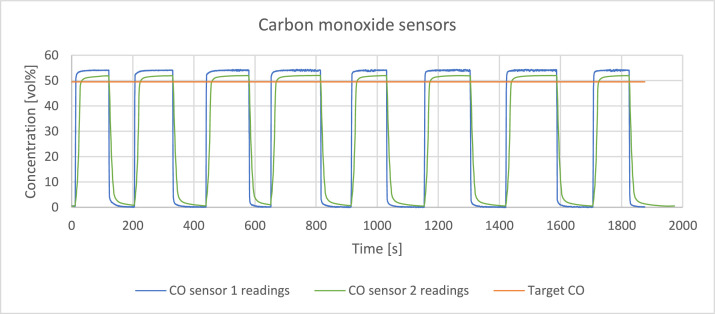
Response of the carbon monoxide sensors to the reference gas mixture.

**Fig. 7:**
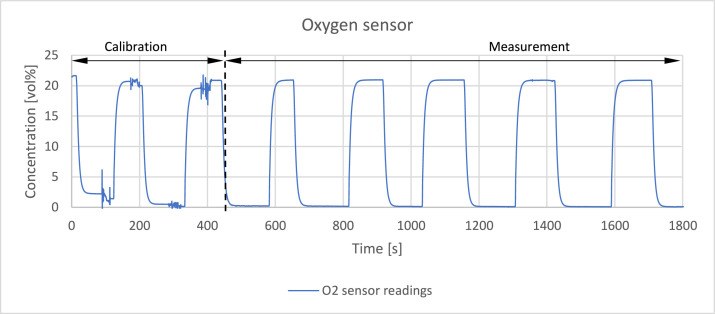
Calibration and response of the oxygen sensor exposed to air. First two plateau and trough correspond to span and zero calibration respectively.

The readings of the sensors were verified by passing a reference gas through the system with a volume flowrate of 0,4 l/min. The reference gas was a mixture of hydrogen, methane, carbon monoxide and carbon dioxide which detailed composition is presented in Table 2. Fig. 3, Fig. 4, Fig. 5, Fig. 6, Fig. 7 present the readings of the single-gas sensors throughout the test. The subsequent pluses of air and the reference gas were supplied to the system by switching the electro valve, initially depicted in Fig. 1, to verify the repeatability of the measurement. During each pulse, which is associated with a peak on the measurement curve, the sensor should give the response corresponding to the concentration of the target gas. A trough on a curve is associated with a pulse of air. It holds for all cases except for oxygen sensor’s readings. Tabulated results of each measurement (pulse) for each gas are presented in Table 3, Table 4, Table 5, Table 6, Table 7.

**Table 2:** The composition of the reference gas mixture.

Gas Concentration [vol%]			
H_2_	CO_2_	CH_4_	CO
35,4 ± 0,22	10,14 ± 0,11	4,95 ± 0,067	49,51 ± 0,397

**Table 3:** Tabulated results of measurements with the hydrogen sensor.

Measurement:	I	II	III	IV	V	VI	VII	VIII	σ
Average Conc [vol%]	34,74	34,70	34,70	34,71	34,76	34,69	34,62	34,64	0,045
Target [vol%]	35,4	35,4	35,4	35,4	35,4	35,4	35,4	35,4	
Absolute error	0,66	0,70	0,70	0,69	0,64	0,71	0,78	0,76	
Relative error	1,86 %	1,99 %	1,99 %	1,95 %	1,82 %	2,00 %	2,19 %	2,15 %

**Table 4:** Tabulated results of measurements with methane sensor.

Measurement:	I	II	III	IV	V	VI	VII	VIII	σ
Average Conc [vol%]	5,24	5,24	5,24	5,23	5,23	5,24	5,24	5,24	0,003
Target [vol%]	4,95	4,95	4,95	4,95	4,95	4,95	4,95	4,95	
Absolute error	0,29	0,29	0,29	0,28	0,28	0,29	0,29	0,29	
Relative error	5,88 %	5,88 %	5,85 %	5,73 %	5,74 %	5,77 %	5,80 %	5,89 %

**Table 5:** Tabulated results of measurements with carbon dioxide sensors.

Sensor	Measurement:	I	II	III	IV	V	VI	VII	VIII	σ
CO_2_ sensor 1	Average Conc [vol%]	13,58	13,55	13,53	13,52	13,49	13,49	13,49	13,44	0,041
	Target [vol%]	10,11	10,11	10,11	10,11	10,11	10,11	10,11	10,11	
	Absolute error	3,47	3,44	3,42	3,41	3,38	3,38	3,38	3,33	
	Relative error	34,27 %	33,99 %	33,82 %	33,70 %	33,46 %	33,40 %	33,42 %	32,98 %	
CO_2_ sensor 2	Average Conc [vol%]	11,10	11,14	11,13	11,18	11,12	11,14	11,15	11,14	0,022
	Target [vol%]	10,14	10,14	10,14	10,14	10,14	10,14	10,14	10,14	
	Absolute error	0,96	1,00	0,99	1,04	0,98	1,00	1,01	1,00	
	Relative error	9,46 %	9,88 %	9,77 %	10,24 %	9,68 %	9,89 %	9,93 %	9,86 %

**Table 6:** Tabulated results of measurements with carbon monoxide sensors.

Sensor	Measurement:	I	II	III	IV	V	VI	VII	VIII	σ
CO sensor 1	Average Conc [vol%]	54,02	54,03	54,06	54,05	53,98	54,07	54,10	54,06	0,038
	Target [vol%]	49,51	49,51	49,51	49,51	49,51	49,51	49,51	49,51	
	Absolute error	4,51	4,52	4,55	4,54	4,47	4,56	4,59	4,55	
	Relative error	9,10 %	9,13 %	9,20 %	9,17 %	9,03 %	9,21 %	9,28 %	9,19 %	
CO sensor 2	Average Conc [vol%]	51,80	51,84	51,91	51,97	51,86	51,96	51,97	51,89	0,064
	Target [vol%]	49,51	49,51	49,51	49,51	49,51	49,51	49,51	49,51	
	Absolute error	2,29	2,33	2,40	2,46	2,35	2,45	2,46	2,38	
	Relative error	4,62 %	4,71 %	4,84 %	4,98 %	4,74 %	4,94 %	4,96 %	4,81 %

**Table 7:** Tabulated results of measurements with oxygen sensor.

Measurement:	I	II	III	IV	V	σ
Average Conc [vol%]	20,91	20,95	20,93	20,88	20,89	0,031

The repeatability of the measurement was assessed by calculating the standard deviation, according to Eq. (1), assuming that each peak is a separate measurement.

(1) $$\sigma =\sqrt{\frac{\sum {\left(x-u\right)}^{2}}{n-1}}$$

Where: x – data values which is the average concentration attained during a peak/plateau (once the signal becomes stable), u – mean of all data values (peaks), n – number of samples (peaks).

The stability of the signal was assessed visually based on the derivative of the sensor’s reading. A signal was considered stable when the value of its derivative was max. twice the derivative values coming from the noise while reading on air.

Hydrogen sensor exhibited both good repeatability and high accuracy of the measurement, surpassing the statistical accuracy provided by the producer (±2vol%). The t90 response time is higher than claimed in the datasheet of the sensor - 25 s instead of <5 s. The potential reason is the presence of the flow adaptor which features a porous wall separating the line from the sensor. The need for gas to diffuse from the line to the sensing cell results in longer response time.

Methane sensor showed an excellent repeatability throughout the test, as well as very fast response time – <2 s. However, there is a slight persisting shift by almost 6 % from the target concentration. It could be due to a cross-sensitivity of the sensor towards other gases, especially CO and CO_2_ which also absorb part of the infra-red spectrum.

CO_2_ sensor 1 exhibited large shift from the reference value which might come from the cross-sensitivity towards other gases. CO_2_ sensor 2 readings have lower relative error but it’s a trade-off between accuracy and longer response time - around 40 s. CO_2_ sensor 1 has much faster response time – <2 s. Both sensors showed good repeatability of measurements.

Carbon monoxide sensors have a slight shift from the target value. CO_2_ sensor 2 has smaller relative error comparing to his faster counterpart – CO_2_ sensor 1. It is paid by longer response time t90 - 13 s versus 2 s.

The zero and span calibration of the oxygen sensor was conducted at the beginning of the experiment. It can be observed during first two full plateau and troughs of the curve in Fig. 7. The calibration gas was reference gas mixture, which composition is presented in Table 2, and air for zero and span calibration respectively. Last five plateau can be regarded as measurements for the sake of repeatability verification which happen to be high based on the value of standard deviation. However, they cannot be used to evaluate the accuracy of the measurement as the sensor’s readings were literally adjusted to the target value. The accuracy of the oxygen sensor is evaluated only in the second part of the methods validation i. e. comparison with results obtained with micro gas chromatography (µGC). Response time of the sensor is slightly higher than claimed in the datasheet – around 10 s.

### Comparison with µGC

Three gas samples were collected from a green sand mold into Tedlar Bags. Reclaimed sand, provided by industrial partner Safe Metal, mixed with bentonite and water were used for moulding. High sand humidity of 5,6 % was achieved to promote high gas evolution rate within the mold. Low carbon steel was poured in the mold at the pouring temperature of 1630 °C. The gas was drawn using Artshu MPA2002T2 pump during 10 min from pouring. Gas sample was dried by passing through a tube filled with silica gel prior to being collected in Tedlar bags. The gas was analysed with Micro GC 3000 (SRA Instruments) at Laboratoire de Multimatériaux et Interfaces (LMI) at University of Lyon prior to using FAGAS. The sampling and analysis were conducted on the same day to prevent any alterations of the composition within samples. Concentration of calibration gases (balance nitrogen) used for quantitative µGC analysis are presented in the Table 8. Concentrations in mol% was converted to vol% assuming ideal gas behaviour.

**Table 8:** Calibration gases used for quantitative µGC. Balance nitrogen.

BOTTLE 1	BOTTLE 2		BOTTLE 3	BOTTLE 4
H_2_	CO_2_	CO	CH_4_	O_2_
1995 ± 40 Mol ppm	2,0059 ± 0,004 Mol%	1,0019 ± 0,002 Mol%	535 ± 11 Mol ppm	20 ± 1 Mol%

**Fig. 8:**
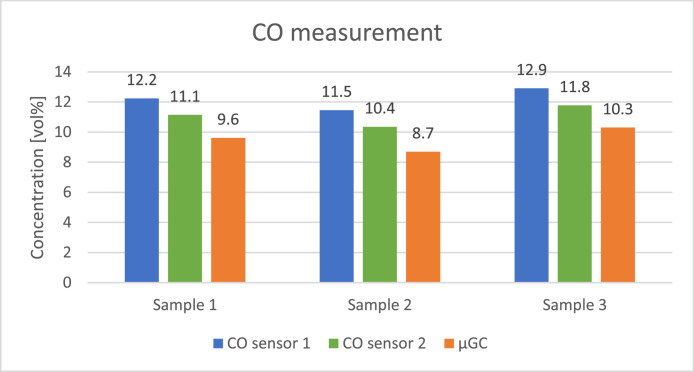
Comparison between results of carbon monoxide quantification using single-gas sensors and µGC.

**Fig. 9:**
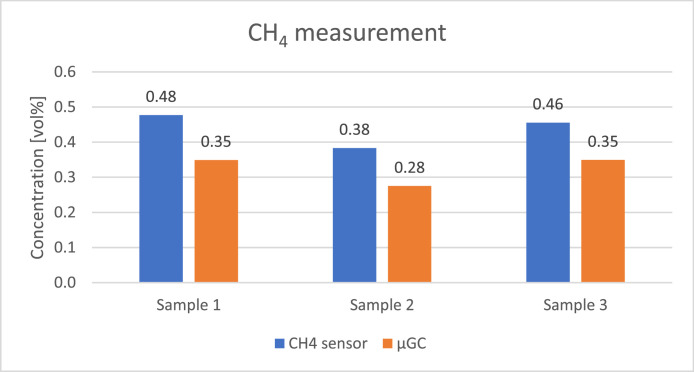
Comparison between results of methane quantification using single-gas sensor and µGC.

**Fig. 10:**
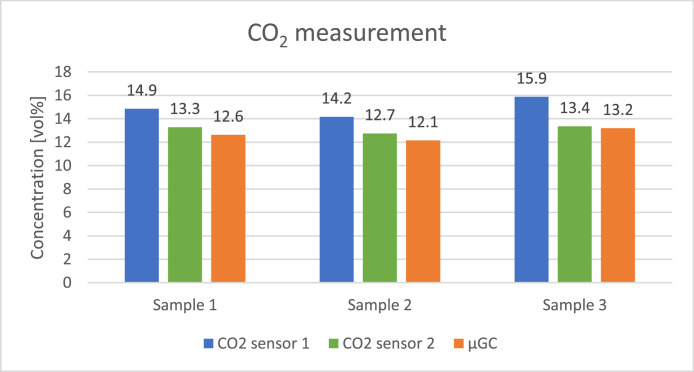
Comparison between results of carbon dioxide quantification using single-gas sensors and µGC.

**Fig. 11:**
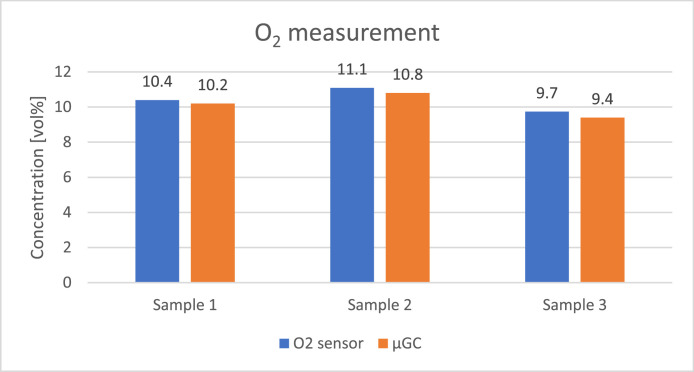
Comparison between results of oxygen quantification using single-gas sensors and µGC.

**Fig. 12:**
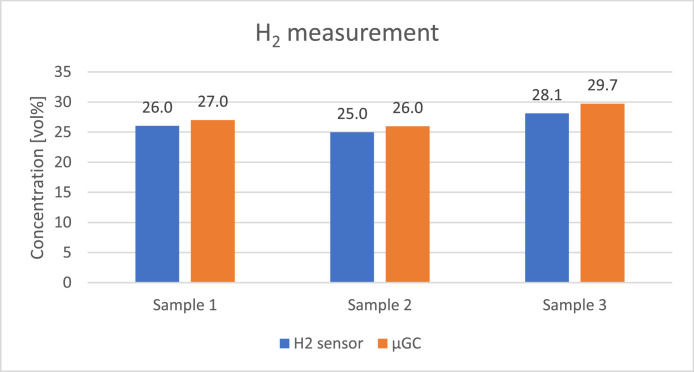
Comparison between results of hydrogen quantification using single-gas sensor and µGC.

The concentration of the gases was calculated assuming it is related linearly to the response of the sensors placed at the end of each column. The measurement was repeated four times on each sample to assure repeatability of the analysis and the corresponding standard deviations are presented in Table 9. Fig. 8, Fig. 9, Fig. 10, Fig. 11, Fig. 12 presents the comparison between results of analysis realised with sensors and µGC. There was just enough gas to make one pulse measurement with FAGAS therefore there is no information on repeatability. Relative error, calculated with respect to concentrations measured with µGC are presented in Table 10.

**Table 9:** Standard deviation of the µGC measurements.

Standard deviation σ [vol%]					
	H_2_	CO_2_	CH_4_	CO	O_2_
Sample 1	0,036	0,019	0,001	0,039	0,037
Sample 2	0,074	0,026	0,001	0,018	0,160
Sample 3	0,244	0,320	0,003	0,053	0,174

**Table 10:** Sensors' relative error with respect to µGC measurements.

Relative error							
Gas:	H_2_	CO_2_		CH_4_	CO		O_2_
Sensor:	H_2_ sensor	CO_2_ sensor 2	CO_2_ sensor 1	CH_4_ sensor	CO sensor 2	CO sensor 1	O_2_ sensor
Sample 1	3,6 %	5,2 %	17,6 %	36,7 %	15,9 %	27,3 %	2,0 %
Sample 2	3,8 %	4,9 %	16,6 %	39,3 %	19,1 %	31,8 %	2,7 %
Sample 3	5,5 %	1,2 %	20,3 %	30,5 %	14,3 %	25,3 %	3,6 %

Levels of hydrogen measured with the H_2_ sensor are well correlated with reference values. Relative error is slightly higher than in case of validation with reference gas mixture. It can be explained by the fact that hydrogen concentration in the bottle used for calibrating µGC was much lower than the hydrogen concentration in the sample. Oxygen sensor also showed a good correlation with µGC measurements. In contrary, relative error of the methane sensor is significant but it should be noted that the concentration of the gas was very low comparing to the measuring range of the sensor (0–15 %vol). CO sensor 1 gave more accurate readings of CO levels than its faster counterpart but for both the relative error was higher than in case of verification with reference gas mixture. The opposite can be observed with carbon dioxide sensors where relative errors of CO_2_ sensor 2 and CO_2_ sensor 1 are smaller compared to those during verification with reference gas mixture with the latter giving relatively reliable readings.

## Limitations

Besides the obvious limitations coming from the measurement range of sensors and the errors in readings which are demonstrated in the previous section there is a limitation coming from the minimal volume of the gas for the testing. If the stable results are to be expected from the CO_2_ sensor 2 the minimal volume of gas required is 1l. This imposes constraint on casting – its geometry and size should such the minimal amounts of gas is generated, in the course of sampling, required for the analysis. As far as continous sampling from the mold is concerned CO_2_ sensor 2 response time is too slow therefore only readings from CO_2_ sensor 1 are viable.

## Conclusion and future work

The created system is capable of gas analysis, although non-negligible errors in readings are presents especially in case of CO and CO_2_ sensors. Nonetheless FAGAS can be used for comparative studies to investigate the influence of process parameters on tendencies in the formation of the atmosphere in sand molds. The future work will focus on creating correction functions for carbon monoxide and carbon dioxide functions by testing gas mixtures of varying compositions. If corrections can be achieved for the readings of thermal conductivity sensors the minimal amount of gas for the analysis would be greatly reduced and the precision of the analysis during continuous gas sampling form the mold greatly improved.

## Ethics statements

The method discussed in this scientific article did not involve human subjects, animal experiments, or data collected from social media platforms.

## Declaration of competing interest

The authors declare that they have no known competing financial interests or personal relationships that could have appeared to influence the work reported in this paper.

## Data Availability

Data will be made available on request.
